# METHODOLOGICAL STRATEGIES AND CHALLENGES FOR EVALUATING PARTICIPANT VIEWS ON DEMENTIA RESEARCH RECRUITMENT

**DOI:** 10.1093/geroni/igz038.2595

**Published:** 2019-11-08

**Authors:** Laura Block, Shannon Mullen, Gay Thomas, Betty Kaiser, Andrea Gilmore-Bykovskyi

**Affiliations:** University of Wisconsin-Madison School of Nursing, Madison, Wisconsin, United States

## Abstract

There is an urgent need to expand enrollment in clinical Alzheimer’s disease (AD) research. Current recruitment methods for AD research predominantly identify patients from primary/specialty clinic settings, potentially creating barriers for individuals unconnected to care. In response to these challenges, the 2018 National Strategy for Research Recruitment and Participation has called for the development of an applied science of recruitment to inform best strategies. However, progress in this area is hindered by methodological challenges to accurately measure AD patient/caregiver participant views on research participation. The objective of this presentation is to report methodological strategies developed to during a prospective qualitative study to investigate AD patient/caregiver views on acute care research recruitment and participation. Participants included patients with dementia (N=2) who had recently been hospitalized and/or their informal caregivers (N=15). We engaged in iterative development and revision of data collection approaches (i.e. semi-structured questions, audiovisual tools, interview guidance) through collaboration with a Community Advisory Board (CAB). Detailed memos were generated to document interview-related challenges, successes and revisions. Therapeutic misconception in delineating research from clinical care was common during interviews regardless of prior research participation. Interview strategies that focus on lived experiences, remove ambiguity from hypothetical recruitment scenarios, and incorporate supportive visual tools that clarify processes around recruitment improved data collection. Challenges included the lack of a common, shared language around recruitment, which was addressed through CAB guidance and input. In conclusion, thoughtful collaboration with community/lay advisers can successfully inform and data collection methods used in applied recruitment research.

